# Notes and Notices

**Published:** 1885-01

**Authors:** 


### NOTES AND NOTICES.

SUPPLEMENT No. 6.—Joslin's Therapeutics of Cholera, with Repertory, will be ready for mailing January 15th.

ERRATUM.—In our December ('84) issue, bottom of page 360 and top of page 361, for *chronic* spasms of cheek, read *clonic* spasms, etc.

PARTNERSHIP.—Dr. C. W. Breyfogle, San Jose, California, has associated with himself Dr. R. E. Pierce, late of Boston.

FOR SALE.—A copy of Teste's *Materia Medica.* Good as new. Price, $3.00.

BEWARE OF TOBACCO.—Lieutenant Greely says that of his nineteen men who perished all but one were smokers, and that one was the last to die. The seven survivors were non-smoking men.

A CONSOLATION.—"The world all praze the philosophers, but toss their pennys into the caps of the monkeys."—JOSH BILLINGS. All doctors who fail to secure the "pennies" can take this wise saying to heart!

DR. W. H. ROMIG.—Dr. William H. Romig, one of the leading physicians in the Lehigh Valley, died December 10th, 1884, of an affection of the heart, after an illness of nearly six weeks. He was a graduate of the University of Pennsylvania and Hahnemann Medical College. After graduation he began practice with his father, Dr. John Romig, in this city, and achieved marked success. His father, who is eighty-one years old, was one of the pioneer homœopathists and retired from active practice about eight years ago. His sons, Dr. W. H. and George, succeeded him.

DR. BERNARD died at Mons, Belgium, 8th of October, 1884. He is said to have died "practically of a broken heart," due to the rapid loss of father, wife, and children—losses sufficient to kill any one. Dr. Bernard was an able man, a frequent writer for the *Revue Homœopathique Belge,* and the author of several works. His book on the treatment of *Constipation* has been translated and reprinted in this country.

THE SANITARY CONFERENCE.—At the session of the National Conference of Health Boards the delegates from the various cities presented reports showing the sanitary condition of the cities and gave their views as to the best means of preventing the introduction of the cholera. Dr. Carson, of Kentucky, made a special report upon the peculiar contagion in West Virginia. He went where the disease was most prevalent in Eastern Kentucky and procured samples of the drinking water. The geological formation forbade the assumption of mineral poison in the water. The streams and ponds had dried down to mere beds of malarial poison. The disease was really epidemic dysentery, caused by malarial poison, and many patients died because the people did not believe in doctors, and called them, if at all, too late. The total number of deaths did not exceed two hundred and twenty-five in Kentucky. The people were deeply aggrieved by the publication of the exaggerated reports of the disease.

The committees on State and municipal action reported upon the best modes of resisting infection. That of the latter recommended, as municipal regulations, that all surface wells be closed, privy vaults abolished, stagnant pools disinfected, receivers kept clear, accumulations of filth prevented in tenements, the food supply inspected, garbage promptly removed, and that the attention of authorities of all institutions, public and private, and of individuals as well, be drawn to the great importance of maintaining habits of personal cleanliness as one of the most efficient means of warding off an attack of cholera or reducing its virulence.

The unanimous opinion of these sanitarians seemed to be that there is every reason to fear that cholera will invade our shores next summer.

